# Asthma in patients hospitalized with pandemic influenza A(H1N1)pdm09 virus infection–United States, 2009

**DOI:** 10.1186/1471-2334-13-57

**Published:** 2013-01-31

**Authors:** Anna M Bramley, Jacek Skarbinski, Alicia M Fry, Lyn Finelli, Seema Jain

**Affiliations:** 1 Influenza Division, Centers for Disease Control and Prevention Atlanta, Atlanta, GA, USA; 2 The CDC Experience Applied Epidemiology Fellow, Atlanta, GA, USA; 3 Present address: 1929 Huxley St., Madison, WI, 53704, USA

**Keywords:** Asthma, Influenza A virus, Hospitalization, ICU, H1N1pdm09

## Abstract

**Background:**

Asthma was the most common co-morbidity among patients hospitalized with pandemic influenza A(H1N1)pdm09 [pH1N1] infection. The objective was to compare characteristics of hospitalized pH1N1 patients with and without asthma and assess factors associated with severity among asthma patients.

**Methods:**

Patient data were derived from two 2009 pandemic case-series of U.S. pH1N1 hospitalizations. A case was defined as a person ≥ 2 years old hospitalized with laboratory-confirmed pH1N1. Asthma status was determined through chart review.

**Results:**

Among 473 cases, 29% had asthma. Persons with asthma were more likely to be 2–17 years old (39% vs. 30%, p = 0.04) and black (29% vs. 18%, p < 0.01), and have chronic obstructive pulmonary disease (13% vs. 9%, p = 0.04) but less likely to have pneumonia (37% vs. 47%, p = 0.05), need mechanical ventilation (13% vs. 23%, p = 0.02), and die (4% vs. 10%, p = 0.04) than those without asthma. Among patients with asthma, those admitted to an intensive care unit (ICU) or who died (n = 38) compared with survivors not admitted to an ICU (n = 99) were more likely to have pneumonia on admission (60% vs. 27%, p < 0.01) or acute respiratory distress syndrome (24% vs. 0%, p < 0.01) and less likely to receive influenza antiviral agents ≤ 2 days of admission (73% vs. 92%, p = 0.02).

**Conclusions:**

The majority of persons with asthma had an uncomplicated course; however, severe disease, including ICU admission and death, occurred in asthma patients who presented with pneumonia. Influenza antiviral agents should be started early in hospitalized patients with suspected influenza, including those with asthma.

## Background

Worldwide, annual influenza epidemics result in an estimated 3–5 million cases of severe illness and 250,000-500,000 deaths every year [1]. Globally, an estimated 300 million people suffer from asthma [2]. Asthma prevalence varies widely between different countries ranging between 1 and 18% [3]. In 2009, the estimated asthma prevalence in the United States was 8% [4]. Previous studies in the U.S. have shown that asthma is common among patients hospitalized with influenza. Among children younger than 18-years-old hospitalized with seasonal influenza from 2003 to 2008, 18% had asthma [5], and among adults aged 18–49 years hospitalized with seasonal influenza from 2005 to 2008, 27% had asthma [6]. In addition, during the 2009 influenza A(H1N1)pdm09 [pH1N1] pandemic, asthma was one of the most common underlying medical conditions among patients hospitalized with pH1N1 infection in the U.S. [7-9] and worldwide [10-15]. In a global pooled analysis looking at risk factors for severe outcomes following pH1N1 infection, asthma was associated with hospitalization and death, but among patients who were hospitalized, those with asthma survived compared with patients with other conditions [15]. Given the burden of asthma among patients hospitalized with pH1N1 infection in the U.S., we sought to better understand patients with asthma and in comparison with patients without asthma through two national case-series conducted during the 2009 pH1N1 pandemic.

## Methods

### Objectives

We describe epidemiological and clinical characteristics of patients with asthma hospitalized with pH1N1 infection in the U.S. during the spring and fall of 2009.

### Patients

Patient data were obtained from two previously described national pH1N1 hospitalizations case-series conducted in the U.S. during the spring [7] and fall [8] of 2009. Patients included in these case-series had laboratory-confirmed pH1N1 virus by real-time reverse-transcriptase polymerase-chain-reaction; diagnostic testing for pH1N1 virus was clinically driven. In the spring (May 1-June 9, 2009), the first 272 hospitalized pH1N1 patients reported to the Centers for Disease Control and Prevention (CDC) were included; participation from 24 states yielded 25% of the total number of cases reported during the spring surveillance period [7]. In the fall (September 1-October 31, 2009), 255 hospitalized pH1N1 patients were sampled from all cases reported based on probability of selection proportional to the number of hospitalized cases reported to CDC; participation from 40 states yielded < 2% of the total number of cases reported to CDC during the fall surveillance period [8].

### Ethics statement

Both spring and fall case-series were part of the emergency public health practice response to assess illness severity during the 2009 pH1N1 pandemic and were deemed not to be research in accordance with the federal human subjects protection regulations at 45 Code of Federal Regulations 46.101c and 46.102d and CDC's Guidelines for Defining Public Health Research and Public Health Non-Research, and therefore were not subject to Institutional Review Board (IRB) review and approval; participation by the state and local health departments was voluntary.

### Study design

For this analysis, we excluded 54 children under two years old because asthma is difficult to diagnose in this age group [16,17]. Demographic and clinical information was abstracted from medical charts by infection control practitioners, physicians, nurses, or epidemiologists at local and state public health departments using a standard clinical form and reported to the CDC.

Day of hospital admission was considered hospital day 0; for transfer patients, date of hospital admission related to the first hospitalization was used. Asthma status was determined by review of the admission history, problem list, and discharge summary. If any of these areas of the medical chart had a history of asthma listed, then the patient was considered to have asthma. Pneumonia status was based on admission chest radiograph reports.

### Statistical analysis

We conducted bivariate analyses to compare characteristics of patients with and without asthma and assessed factors associated with intensive care unit (ICU) admission and death among patients with asthma. We stratified all categorical bivariate analyses by the age groups 2–9 years, 10–17 years, 18–49 years and ≥50 years and used either the chi-square or Fisher’s Exact test to compare categorical variables and the Wilcoxon rank-sum test to compare continuous variables (P ≤0.05). Data were analyzed using SAS version 9.2 (SAS Institute, Cary, NC).

## Results

Among 473 patients two years or older hospitalized with pH1N1 infection in the two case-series, 137 (29%) had asthma; this included 74/249 (30%) of spring and 63/224 (28%) of fall hospitalizations. Thirty-five per cent (54/154) of children aged 2 to 17 years and 26% (83/319) of adults aged 18 years or older had asthma. Patients with asthma were significantly more likely than those without asthma to be 2 to17 years old (39% versus 30%, p = 0.04), and to be black (29% versus 18%, p < 0.01) (Table 1).

**Table 1:** Demographic characteristics: comparison of patients with and without asthma (n = 473)

**Characteristic**	**Patients with asthma**	**Patients without asthma**	*P-*Value^*^
	**No. (%) (n = 137)**	**No. (%) (n** = **336)**	
Female	81 (59)	175/334 (52)	0.07
Median age in years (range)	27 (3 – 80)	30 (2 – 87)	0.15
Age 2–17 years	54 (39)	100 (30)	0.04
Age group			0.08
2–9 years	28 (20)	54 (16)	0.26
10–17 years	26 (19)	46 (14)	0.15
18–49 years	58 (42)	160 (48)	0.30
≥50 years	25(18)	76 (23)	0.29
Race and ethnicity			0.17
Non-Hispanic White	47 (34)	137 (41)	0.19
Black	40 (29)	60 (18)	<0.01
Hispanic	28 (20)	78 (23)	0.51
Unspecified	15 (11)	40 (12)	0.77
Other^†^	7 (4)	18 (5)	0.91

*P-Values stratified by age-group.

^†^Other race and ethnicity includes Asian, Native Hawaiian or Pacific Islander, American Indian or Alaska Native and Multiracial.

Patients with asthma were not more likely to be admitted within 2 days of illness onset than patients without asthma (54% versus 45%, p = 0.06); however, in the 10–17 year old age group, patients with asthma were more likely to be admitted within 2 days of illness onset (77% versus 51%, p = 0.03) (Table 2). Length of hospital stay was similar between the two groups. Patients with asthma were significantly more likely to present with shortness of breath and wheezing than those without asthma. Patients with asthma were significantly more likely to have chronic obstructive pulmonary disease (COPD) (13% versus 9%, p = 0.04) and were less likely to have chronic renal disease (2% versus 11%, p < 0.01) than patients without asthma. Of the patients with asthma who also had COPD, 9/16 (56%) were current smokers; former smoking status was not available. Patients with asthma were less likely than those without asthma to have radiographic findings consistent with pneumonia (37% versus 47%, p = 0.05). There was no statistical difference in positive sterile site bacterial culture (1% versus 4%, p = 0.11) confirmed by admission blood culture, sterile respiratory site culture, or urine antigen test between the two groups.

**Table 2:** Clinical characteristics: comparison of patients with and without asthma (n = 473)

**Characteristic**	**Patients with asthma**	**Patients without asthma**	*P-*Value^*^
	**No. (%) (n = 137)**	**No. (%) (n = 336)**	
Median days from onset to admission (range)	2 (0 – 20) (n = 136)	3 (0 – 21) (n = 332)	0.06
Admission within 2 days of illness onset	74/136 (54)	150/334 (45)	0.06
Median length of stay- days (range)	3 (0 – 47) (n = 136)	3 (0 – 77) (n = 332)	0.14
Clinical symptoms at admission			
Cough	118 (86)	284 (85)	0.45
Fever	115 (84)	294 (88)	0.20
Shortness of Breath	91 (66)	155 (46)	<0.01
Wheezing	62 (45)	47 (14)	<0.01
Fatigue/Weakness	47 (34)	125 (37)	0.71
Myalgias	43 (31)	122 (36)	0.57
Rhinorrhea	42 (31)	103 (31)	0.88
Chills	38 (28)	120 (36)	0.23
Sore Throat	33 (24)	96 (29)	0.25
Vomiting	31 (23)	98 (29)	0.08
Headache	31 (23)	99 (30)	0.17
Diarrhea	18 (13)	75 (22)	0.03
Underlying medical condition			
Any condition excluding asthma	64 (47)	217 (50)	0.11
Diabetes	24 (18)	51 (15)	0.18
Chronic obstructive pulmonary disease	18 (13)	31 (9)	0.04
Cardiovascular disease	16 (12)	52 (16)	0.55
Immunosuppression	15 (11)	48 (14)	0.45
Neuromuscular disorder	11 (8)	26 (8)	0.87
Cognitive dysfunction	10 (7)	25 (7)	0.76
Seizure disorder	7 (5)	25 (7)	0.21
Chronic renal disease	3 (2)	38 (11)	<0.01
Pregnancy	8 (6)	38 (12)	0.11
Current smoker	23/121 (19)	58/303 (19)	0.56
Obese or morbidly obese^†^	47/129 (36)	103/298 (35)	0.37
Positive sterile site culture	1/137 (1)	13/336 (4)	0.11
Pneumonia at admission	45/123 (37)	133/281 (47)	0.05
Seasonal flu vaccine	33/86 (38)	64/203 (32)	0.49

^*^P-Values stratified by age-group.

^†^Body-Mass Index (BMI) was calculated for a subset of patients for whom height and weight were available to determine obesity (BMI 30–39.9 in adults ≥18 years or BMI percentile 95–100 in children 2 to 19 years old) and morbid obesity (BMI ≥40 in adults only); pregnant women were excluded from this calculation.

There were no significant differences in the proportion of ICU admission (28% versus 34%) or diagnosis of acute respiratory distress syndrome (ARDS) (7% versus 13%) between the two groups (Table 3). However, patients with asthma compared with patients without asthma were significantly less likely to require invasive mechanical ventilation (13% versus 23%, p = 0.02) and to die while hospitalized (4% versus 10%, p = 0.04).

**Table 3:** Clinical characteristics and outcomes: comparison of patients with and without asthma (n = 473)

**Characteristic**	**Patients with asthma**	**Patients without asthma**	*P-*Value^*^
	**No. (%) (n = 137)**	**No. (%) (n = 336)**	
Tachypnea	60/137 (44)	110/336 (33)	0.07
Tachycardia	88/137 (64)	213/336 (63)	0.86
Intensive care unit admission	37/133 (28)	108/321 (34)	0.31
Mechanical ventilation	16/127 (13)	69/301 (23)	0.02
Acute respiratory distress syndrome	9/129 (7)	38/292 (13)	0.10
Sepsis syndrome	7/128 (6)	31/291 (11)	0.12
Death	5/137 (4)	32/332 (10)	0.04
Influenza antiviral agent receipt			
Any during hospitalization	101/137 (74)	270/336 (80)	0.15
≤ 2 days of illness onset	49/99 (50)	106/269 (39)	0.10
≤ 2 days of admission	79/92 (86)	219/253 (87)	0.87
Median days from illness onset to antiviral initiation (range)	3 (0 – 18)	3 (0 – 32)	0.06
Other treatment			
Antibiotics	101/137 (74)	260/336 (77)	0.40
Steroids	83/137 (61)	96/336 (29)	<0.01

^*^P-Values stratified by age-group.

Overall, hospitalized patients with and without asthma were equally likely to receive influenza antiviral agents (74% versus 80%) (Table 3). There was also no significant difference between the two groups in receipt of antiviral agents within 2 days of illness onset (50% versus 39%) or within 2 days of admission (86% versus 87%). However, among 72 patients 10–17 years old, patients with asthma were more likely than patients without asthma to receive antiviral agents within 2 days of illness onset (89% versus 38%, p < 0.01) and within 2 days of admission (100% versus 76%, p = 0.03). Patients with asthma were significantly more likely than those without asthma to receive oral or intravenous steroids (61% versus 29%, p < 0.01).

Among patients hospitalized with pH1N1 infection who had asthma, those admitted to an ICU or who died (n = 38) compared with survivors not admitted to an ICU (n = 99) were significantly more likely to present with tachypnea, have pneumonia, ARDS, and a longer median length of hospital stay (6 days versus 2 days) (Table 4). Patients with asthma who were admitted to an ICU or died compared with survivors who were not admitted to an ICU were equally likely to have an underlying medical condition other than asthma. Patients with asthma who were admitted to an ICU or died were equally likely to receive antiviral agents compared with survivors who were not admitted to an ICU, including within 2 days of illness onset, but were significantly less likely to receive antiviral agents within 2 days of admission (73% versus 92%, p = 0.02).

**Table 4:** Characteristics for ICU admission and death among patients with asthma hospitalized with 2009 Pandemic Influenza A (H1N1) Infection: comparison of patients with asthma admitted to an ICU or who died versus patients with asthma who were not admitted to an ICU and survived (n = 137)

**Characteristic**	**Intensive care unit or deaths**	**Non-intensive care unit survivors**	*P-*Value^*^
	**No. (%) (n = 38)**	**No. (%) (n = 99)**	
Median days from onset to admission (range)	2 (0 – 15)	2 (0 – 20)	0.52
Admission within 2 days of illness onset	20 (53)	54 (55)	0.80
Median length of stay- days (range)	6 (0 – 47)	2 (1 – 10)	<0.01
Age 2–17 years old	12/38 (32)	42/99 (42)	0.24
Underlying medical condition excluding asthma	20 (53)	44 (44)	0.39
Pregnancy	3 (8)	5 (5)	0.83
Current smoker	7 (22)	16 (18)	0.82
Obese or morbidly obese^†^	8/29 (28)	21/69 (30)	0.29
Tachypnea	23/38 (61)	37/99 (37)	<0.01
Tachycardia	28/38 (74)	60/99 (60)	0.12
Mechanical ventilation	16/38 (42)	0/99 (0)	<0.01
Acute respiratory distress syndrome	9/38 (24)	0/99 (0)	<0.01
Sepsis syndrome	6/38 (16)	0/99 (0)	<0.01
Pneumonia at admission	22/37 (60)	23/86 (27)	<0.01
Seasonal flu vaccination	8/25 (32)	23/29 (39)	0.45
Influenza antiviral agent receipt			
Any during hospitalization	32/38 (84)	68/99 (69)	0.07
≤2 days of illness onset	13/32 (41)	36/66 (55)	0.14
≤2 days of admission	22/30 (73)	57/62 (92)	0.02
Median days from illness onset to antiviral initiation (range)	3 (0 – 15)	2 (0 – 15)	0.15
Other treatment			
Antibiotics	35/38 (92)	64/99 (65)	<0.01
Steroids	25/38 (66)	58/99 (59)	0.45

^*^P-Values stratified by age-group.

^†^Body-Mass Index (BMI) was calculated for a subset of patients for whom height and weight were available to determine obesity (BMI 30–39.9 in adults ≥18 years or BMI percentile 95–100 in children 2 to 18 years old) and morbid obesity (BMI ≥40 in adults only); pregnant women were excluded from this calculation.

## Discussion

Among a national case-series of patients hospitalized with pH1N1 infection in the U.S., during both waves of the pH1N1 pandemic, almost one in every three patients hospitalized with pH1N1 virus infection two years or older had asthma. The majority of hospitalized persons with asthma and pH1N1 infection had an uncomplicated hospital course with less pneumonia upon admission, need for mechanical ventilation and death in this group compared with those without asthma. However, almost 40% of the patients hospitalized with pH1N1 infection with asthma had pneumonia upon admission, and these patients were more likely to require ICU admission or die than patients with asthma who did not have pneumonia upon admission. Data from this analysis suggest that early treatment with influenza antiviral agents within 2 days of admission may be beneficial in reducing illness severity in patients with asthma.

In our analysis, the proportion of children (35%) and adults (26%) with asthma who were hospitalized with pH1N1 infection was notably higher than the national prevalence of asthma among children (9.6%) and adults (7.7%) in the U.S. [17]. These data are consistent with both seasonal and pandemic influenza U.S. reports demonstrating similar proportions of asthma among patients hospitalized with influenza. From 2005–2008, among adults (n = 1267) hospitalized with seasonal influenza, 27% of adults aged 18–49 years had asthma [6]. In a recent national multi-center study that included 2,165 children aged 2–17 years old hospitalized with influenza from 2003 to 2009, 32% had asthma, with a higher proportion (44%) during the 2009 pH1N1 pandemic compared with previous seasons [18]. Data from international studies reported similar proportions of asthma among hospitalized patients with pH1N1 infection including reports from Australia (31%), Ireland (18%), Singapore (19%), Spain (23%), and the United Kingdom (25%) [10-14].

In our age group stratified analysis, patients with asthma were more likely to have COPD than patients without asthma. As over 20% of the patients in our case-series were 50 years or older and there is an overlap in asthma and COPD obstructive pathophysiology in the airways [19], this is not an unexpected finding. As persons with asthma age, the airway obstruction becomes less reversible due to airway remodeling from chronic inflammation and fibrosis leading to a more chronic obstructive pathology that retains features of asthma [20]. The elderly may have asthma alone, COPD alone, or both; diagnosing either asthma or COPD alone may be challenging, in part because these patients may not be regularly monitored with spirometry to document progression of their obstructive disease [21]. In addition, almost 60% of patients with asthma and COPD were current smokers, which likely contributed to their dual co-morbidities.

Approximately 30% of patients with and without asthma in our analysis were admitted to the ICU, but overall the majority of patients with asthma had an uncomplicated hospital course, including less mechanical ventilation and death compared with those without asthma. The reasons for this are unclear. Patients included in our analysis may have been admitted to an ICU more readily if they had a history of asthma as a precaution and not due to the severity of their present illness; this would bias our analysis to demonstrating less severe outcomes in this group. Other reports which also used chart review to determine presence of asthma have reported similar findings. In a recent national multi-center study that included 1,434 children aged 2–17 years old with asthma who were hospitalized with seasonal and pandemic influenza from 2003 to 2009, 14–24% were admitted to the ICU but only 2–11% had respiratory failure and < 1% of these patients required extracorporeal membrane oxygenation or died [18]. In a California pH1N1 case-series that included 1,088 adults and children, 24% of patients had asthma but fewer deaths occurred in those with asthma than those without asthma (7% versus 12%) [9]. In a global pooled analysis of patients hospitalized with pH1N1 infection that included asthma data from 11 countries, while asthma was the most common underlying condition associated with hospitalization, a higher proportion of patients with asthma survived compared with patients with other conditions [15]. When looking at country specific data, this also holds true, including in one of the largest studied cohorts in the United Kingdom where asthma was the most common underlying condition among 1520 hospitalized pH1N1 patients but had a significant lower odds of death [22].

Patients with asthma in our analysis were also less likely to have a diagnosis of pneumonia upon admission than patients without asthma. Our study results are in contrast with a recent national multi-center study of 2,992 children under 18 years old hospitalized with seasonal influenza from 2003 to 2008 who had a chest radiograph performed, in which patients with asthma were more likely than those without asthma to have pneumonia (41% versus 34%, p < 0.01) [23]. This study differed from our analysis in the following ways: it examined seasonal not pandemic influenza, included only children, and permitted the chest radiograph to be performed anytime during hospitalization as opposed to at admission as is used for our analysis. These study design differences could help explain the different findings between these two studies. However, it is important to note that in both studies, almost 40% of patients with asthma had pneumonia. Pneumonia is a known complication of influenza [24,25] and is an important cause of morbidity during seasonal and pandemic influenza periods [5,23]. Further clarity on the relationship between asthma and influenza-associated pneumonia is needed to better understand which persons with asthma are at greater risk for pneumonia.

The majority of patients in our analysis received influenza antiviral agents with no significant differences among those with and without asthma, including in relation to illness onset or admission time; in addition there was no significant difference in time from illness onset to admission between the two groups. However, only 50% of patients with asthma received antiviral agents within 2 days of illness onset. It is unclear if delayed treatment was due to delay in testing, delayed ascertainment of results, or antiviral agent clinical prescribing practice. When looking at factors associated with ICU admission or death, early antiviral treatment in relation to hospital admission was found to be protective among those with asthma. This adds to the existing evidence underscoring the importance of early influenza treatment among those persons with suspected or confirmed influenza infection who are hospitalized and those with underlying medical conditions, including asthma, regardless of their prior vaccination status as is recommended by the Advisory Committee on Immunization Practices (ACIP) [26].

Our data are subject to limitations. The patients described in this analysis were derived from two hospitalization case-series that used different sampling methods [7,8]. However, data from both periods were nationally representative of hospitalizations from areas in the U. S. where peak disease activity was occurring at the time. Patients included in this analysis were confirmed for pH1N1 virus and may not be representative of hospitalized patients who may not have been tested. Despite use of a standard data collection form, not all information was collected for all patients, including influenza vaccination status (pH1N1 vaccine was not readily available during the study); this limits our ability to assess these interventions, however the study was not designed to address these specific questions. In addition diagnoses of asthma and ARDS were ascertained from history and clinical diagnosis and not by a standardized clinical assessment. We were also not able to determine the level of baseline severity of asthma among patients described in this cases-series, including past hospitalizations, intubations, and steroid or inhaler use, which could help explain which persons with asthma are at risk for more severe outcomes. Patients with asthma may have been misclassified as having COPD or vice versa.

## Conclusions

Among patients hospitalized with pH1N1 virus infection in 2009, asthma was the most common underlying medical condition. While most patients with asthma had an uncomplicated course of illness, severe disease, including ICU admission and death, still occurred, especially in those who had pneumonia on admission. Since 1964, persons with asthma have been prioritized for influenza vaccination by the ACIP; however, they remain under-immunized with less than 30% influenza vaccine coverage in this group [27]. Vaccine is the primary tool for prevention of influenza infection and should continue to be encouraged in patients with asthma. This study also suggests that early antiviral therapy can reduce influenza-associated complications, and should be started as early as possible in all hospitalized patients and in outpatients with high-risk conditions, including those with asthma.

## Abbreviations

pH1N1 = H1N1pdm09: Pandemic influenza A (H1N1) infection; ICU: Intensive Care Unit; CDC: Centers for Disease Control and Prevention; IRB: Institutional Review Board; COPD: Chronic obstructive pulmonary disease; ARDS: Acute respiratory distress syndrome; ACIP: Advisory Committee on Immunization Practices.

## Competing interests

No potential conflict of interest relevant to this article was reported. None of the authors have received reimbursements, fees, funding, or salary from an organization that may in any way have influenced our professional judgment.

## Authors’ contributions

All authors contributed to the conception, study design, and interpretation of data. JM, AB, JS, and SJ acquired and analyzed the data. JM and SJ drafted the manuscript, with substantial involvement from all other authors. All authors read and approved the final version of the manuscript.

## Pre-publication history

The pre-publication history for this paper can be accessed here:

http://www.biomedcentral.com/1471-2334/13/57/prepub
